# Economic evaluation of thermal ablation compared to cryotherapy and loop diathermy in a screen-and-treat approach to cervical cancer, Zambia

**DOI:** 10.2471/BLT.24.292792

**Published:** 2025-07-09

**Authors:** Ahmad Fuady, Charlotte Kasempa, Eric Lucas, Namakau Nyambe, Darcy W Rao, Vanessa Tenet, Nathalie Broutet, Richard Muwonge, Mulindi Mwanahamuntu, Iacopo Baussano, Groesbeck P Parham, Partha Basu

**Affiliations:** a Department of Community Medicine, Universitas Indonesia, Jakarta, Indonesia.; b Department of Obstetrics and Gynaecology, Cancer Diseases Hospital, Lusaka, Zambia.; c Early Detection, Prevention and Infections Branch, International Agency for Research on Cancer (IARC/WHO), 25 avenue Tony Garnier, Lyon, 69007, France.; d Evidence Action, Lusaka, Zambia.; e Gender Equality Division, Bill & Melinda Gates Foundation, Seattle, United States of America (USA).; f Department of Sexual and Reproductive Health and Research, World Health Organization, Geneva, Switzerland.; g Department of Obstetrics & Gynaecology, University of North Carolina, Chapel Hill, USA.; h University Teaching Hospital, Women and Newborn Hospital, Lusaka, Zambia.

## Abstract

**Objective:**

To estimate the financial and economic costs and the cost–effectiveness of thermal ablation compared to cryotherapy and loop diathermy within a screen-and-treat approach to cervical cancer screening in Zambia.

**Methods:**

We analysed costs within a randomized controlled trial in which women eligible for ablative treatment after cervical cancer screening were assigned to one of three treatment arms: thermal ablation, cryotherapy or loop diathermy. We used a microcosting approach to calculate programme, personnel, equipment and consumable costs for two groups: women treated without follow-up (screened-and-treated) and women who completed follow-up (follow-up-completed). We also estimated trial costs and projected costs if the screen-and-treat approach were to be integrated into routine cervical cancer services. To assess how cost-effective the treatments were, we used a decision tree model.

**Findings:**

Out of the 3124 women who were screened-and-treated, 2386 (76.4%) completed follow-up. In the trial scenario, costs for thermal ablation were lower than cryotherapy and loop diathermy, both per screened-and-treated woman (39.6 United States dollars (US$) versus US$ 42.3 and US$ 50.6, respectively) and per follow-up-completed woman (US$ 55.1 versus US$ 57.9 and US$ 66.2, respectively). In the routine scenario, costs for thermal ablation were also lower than for other treatments (US$ 12.7 versus US$ 15.6 and US$ 34.9, respectively, for screen-and-treat) due to significantly lower personnel costs. Thermal ablation was cost-effective compared to cryotherapy and loop diathermy.

**Conclusion:**

Our study suggests that thermal ablation is a cost-effective option for the screen-and-treat approach to cervical cancer screening compared with cryotherapy and loop diathermy.

## Résumé

**Objectif:**

Estimer les coûts financiers et économiques et le rapport coût-efficacité de l’ablation thermique par rapport à la cryothérapie et au traitement par anse diathermique dans le cadre d’une approche de dépistage et de traitement du cancer du col de l’utérus en Zambie.

**Méthodes:**

Nous avons analysé les coûts dans le cadre d’un essai contrôlé et randomisé au cours duquel des femmes éligibles pour un traitement ablatif après un dépistage du cancer du col de l’utérus ont été assignées à l’un des trois groupes de traitement: ablation thermique, cryothérapie ou traitement par anse diathermique. Nous avons utilisé une approche de décomposition précise des coûts pour calculer les coûts du programme, du personnel, de l’équipement et des consommables pour deux groupes: les femmes traitées sans suivi (dépistées et traitées) et les femmes qui ont achevé le suivi (suivi achevé). Nous avons également estimé les coûts de l’essai et les coûts prévus si l’approche de dépistage et de traitement devait être intégrée dans les services de routine pour le cancer du col de l’utérus. Pour évaluer le rapport coût-efficacité des traitements, nous avons utilisé un modèle d’arbre décisionnel.

**Résultats:**

Sur les 3 124 femmes dépistées et traitées, 2 386 (76,4%) ont achevé le suivi. Dans le scénario de l’essai, les coûts de l’ablation thermique étaient inférieurs à ceux de la cryothérapie et au traitement par anse diathermique, à la fois par femme dépistée et traitée (39,6 dollars américains (USD), contre 42,3 et 50,6 USD, respectivement) et par femme ayant achevé le suivi (55,1 USD contre 57,9 et 66,2 USD, respectivement). Dans le scénario de routine, les coûts de l’ablation thermique étaient également inférieurs à ceux des autres traitements (12,7 USD contre 15,6 et 34,9 USD, respectivement, pour le dépistage et le traitement) en raison de coûts de personnel nettement inférieurs. L’ablation thermique était rentable par rapport à la cryothérapie et au traitement par anse diathermique.

**Conclusion:**

Notre étude suggère que l’ablation thermique est une option rentable pour l’approche de dépistage et de traitement du cancer du col de l’utérus par rapport à la cryothérapie et au traitement par anse diathermique.

## Resumen

**Objetivo:**

Estimar los costos financieros y económicos, así como la rentabilidad de la ablación térmica en comparación con la crioterapia y la diatermia con asa, en el marco de una estrategia de cribado y tratamiento del cáncer de cuello uterino en Zambia.

**Métodos:**

Se analizaron los costos en el contexto de un ensayo controlado aleatorizado en el que mujeres elegibles para tratamiento ablativo tras el cribado de cáncer de cuello uterino fueron asignadas a uno de tres grupos de tratamiento: ablación térmica, crioterapia o diatermia con asa. Se utilizó un enfoque de microcosteo para calcular los costos del programa, personal, equipamiento e insumos para dos grupos: mujeres tratadas sin seguimiento posterior (cribado y tratamiento) y mujeres que completaron el seguimiento (seguimiento completado). Asimismo, se estimaron los costos del ensayo y se proyectaron los costos en caso de integrar esta estrategia de cribado y tratamiento en los servicios rutinarios de cáncer de cuello uterino. Para evaluar la rentabilidad de los tratamientos, se empleó un modelo de árbol de decisiones.

**Resultados:**

De las 3124 mujeres tratadas tras el cribado, 2386 (76,4%) completaron el seguimiento. En el escenario del ensayo, los costos de la ablación térmica fueron inferiores a los de la crioterapia y la diatermia con asa, tanto por mujer tratada tras el cribado (39,6 dólares estadounidenses, US$, frente a US$ 42,3 y US$ 50,6, respectivamente) como por mujer que completó el seguimiento (US$ 55,1 frente a US$ 57,9 y US$ 66,2, respectivamente). En el escenario rutinario, los costos de la ablación térmica también fueron inferiores a los de los demás tratamientos (US$ 12,7 frente a US$ 15,6 y US$ 34,9, respectivamente, por cribado y tratamiento), debido a costos de personal significativamente menores. La ablación térmica resultó ser una opción rentable en comparación con la crioterapia y la diatermia con asa.

**Conclusión:**

Los resultados del estudio sugieren que la ablación térmica representa una opción rentable dentro del enfoque de cribado y tratamiento para la detección del cáncer de cuello uterino, en comparación con la crioterapia y la diatermia con asa.

## ملخص

الغرض تقدير التكاليف المالية والاقتصادية، وفعالية تكلفة الاستئصال الحراري مقارنةً بالعلاج بالتبريد، والمعالجة الحرارية الحلقية، في أسلوب الفحص والعلاج لسرطان عنق الرحم في زامبيا.

الطريقة قمنا بتحليل التكاليف في تجربة عشوائية خاضعة للتحكم، حيث تم توزيع النساء المؤهلات للعلاج بالاستئصال بعد فحص سرطان عنق الرحم على أحد ثلاثة أجنحة علاجية: الاستئصال الحراري، أو العلاج بالتبريد، أو المعالجة الحرارية الحلقية. استخدمنا أسلوب التكلفة الجزئية لحساب تكاليف البرنامج، وفرق العمل، والمعدات، والمواد الاستهلاكية لمجموعتين: النساء اللاتي عولجن دون متابعة (خضعن للفحص والعلاج)، والنساء اللاتي أكملن المتابعة (تم إكمال المتابعة). كما قمنا بتقدير تكاليف التجربة والتكاليف المتوقعة في حال دمج أسلوب الفحص والعلاج في خدمات سرطان عنق الرحم الروتينية. لتقييم فعالية العلاجات من حيث التكلفة، استخدمنا نموذج شجرة القرار.

النتائج من بين 3124 امرأة خضعن للفحص والعلاج، أكملت 2386 (%76.4) منهن فترة المتابعة. في سيناريو التجربة، كانت تكاليف الاستئصال الحراري أقل من العلاج بالتبريد والمعالجة الحرارية الحلقية، لكل امرأة خضعت للفحص والعلاج (39.6 دولارًا أمريكيًا، مقابل 42.3 دولارًا أمريكيًا، و50.6 دولارًا أمريكيًا على التوالي) ولكل امرأة أكملت المتابعة (55.1 دولارًا أمريكيًا مقابل 57.9 دولارًا أمريكيًا، و66.2 دولارًا أمريكيًا على التوالي). في السيناريو الروتيني، كانت تكاليف الاستئصال الحراري أيضًا أقل من العلاجات الأخرى (12.7 دولارًا أمريكيًا مقابل 15.6 دولارًا أمريكيًا، و34.9 دولارًا أمريكيًا، على التوالي، للفحص والعلاج) نظرًا لانخفاض تكاليف الموظفين بشكل ملحوظ. كان الاستئصال الحراري فعالًا من حيث التكلفة مقارنةً بالعلاج بالتبريد والمعالجة الحرارية الحلقية.

الاستنتاج تشير دراستنا إلى أن الاستئصال الحراري يعد خيارًا فعالاً من حيث التكلفة لنهج الفحص والعلاج لسرطان عنق الرحم مقارنة بالعلاج بالتبريد والمعالجة الحرارية الحلقية.

## 摘要

目的 旨在评估在赞比亚采用“筛查即治疗”策略以筛查宫颈癌的过程中，与冷冻疗法和环形电切术相比，热消融术的财务和经济成本及其成本效益。

方法 根据宫颈癌筛查结果，我们将符合接受消融治疗条件的女性随机分配至以下三个治疗组之一：热消融术、冷冻疗法或环形电切术，以开展一项随机对照试验，进而分析三种疗法的成本。我们采用微观成本分析法来计算以下两个试验组的项目、人员、设备和耗材成本：接受治疗后无跟踪随访的女性（筛查即治疗）和完成了跟踪随访的女性（完成跟踪随访）。我们还估算了在将“筛查即治疗”策略纳入常规宫颈癌服务的情况下的试验成本和预计成本。我们使用了一个决策树模型来评估不同疗法的成本效益。

结果 在参与“筛查即治疗”计划的 3,124 名女性中，有 2,386 名（占 76.4%）完成了跟踪随访。在试验场景下，热消融术的成本比冷冻疗法和环形电切术的成本都要低，无论是对于仅完成“筛查即治疗”的女性（39.6 美元/人，而另外两种疗法分别为 42.3 美元/人和 50.6 美元/人），还是完成了跟踪随访的女性（55.1 美元/人，而另外两种疗法分别为 57.9 美元/人和 66.2 美元/人）。在常规场景下，由于热消融术的人员成本明显更低，所以该疗法的成本也比其他两种疗法低（对于仅完成“筛查即治疗”的女性：12.7 美元，而另外另种疗法分别为 15.6 美元和 34.9 美元）。与冷冻疗法和环形电切术相比，热消融术更具成本效益。

结论 我们的研究表明，与冷冻疗法和环形电切术相比，在采用“筛查即治疗”策略来筛查宫颈癌时，热消融术是一种颇具成本效益的疗法。

## Резюме

**Цель:**

Оценить финансовые и экономические затраты, а также экономическую эффективность использования термоабляции в сравнении с криотерапией и петлевой диатермией при скрининге и лечении рака шейки матки в Замбии.

**Методы:**

Авторы проанализировали затраты в рамках рандомизированного клинического исследования, где женщинам, которым требовалось абляционное вмешательство по итогам скрининга на рак шейки матки, назначали один из трех видов лечения, а именно: термоабляцию, криотерапию или петлевую диатермию. Был использован метод микрозатрат для расчета затрат на программу, персонал, оборудование и расходные материалы для двух групп: женщин, которые были пролечены без последующего контроля (то есть прошедшие только скрининг и лечение), и женщин, которые прошли последующий контроль (группа с последующим контролем). Были также оценены затраты на исследование и предполагаемые затраты, если бы подход «скрининг и лечение» был интегрирован в регулярные медицинские услуги в связи с раком шейки матки. Для оценки экономической эффективности лечения авторы использовали модель древа решений.

**Результаты:**

Из 3124 женщин, которые прошли скрининг и лечение, 2386 (76,4%) завершили последующий контроль. В сценарии исследования затраты на термоабляцию были ниже, чем на криотерапию и петлевую диатермию, как для женщин, которые прошли только скрининг и лечение (39,6 доллара США против 42,3 и 50,6 доллара США соответственно), так и для женщин, прошедших последующий контроль (55,1 доллара США против 57,9 и 66,2 доллара США соответственно). В сценарии регулярного вмешательства затраты на термоабляцию также были ниже, чем на другие виды вмешательств (12,7 доллара США против соответственно 15,6 и 34,9 доллара США для режима «скрининг и лечение»), вследствие значительно более низких затрат на персонал. Термоабляция показала себя экономически эффективной в сравнении с криотерапией и петлевой диатермией.

**Вывод:**

Исследование показывает, что термоабляция является экономически эффективной опцией при подходе «скрининг и лечение» в случае скрининга на рак шейки матки, если сравнивать с криотерапией и петлевой диатермией.

## Introduction

Screen-and-treat approaches for cervical cancer prevention are widely used in low- and middle-income countries,^1^ in line with World Health Organization (WHO) guidelines.^2^ Offering treatment based on a positive screening test (without histopathological verification) reduces the need for repeated clinic visits, thus saving time and transportation costs and reducing loss-to-follow-up for treatment.^3^^–^^6^ Under the screen-and-treat approach, women are screened either by visual inspection (of the cervix) with acetic acid or by testing for human papillomavirus (HPV). Treatment is offered immediately following a positive test result to reduce the future risk of developing high-grade precancer or cancer.

Zambia has a cervical cancer incidence of 65.5 cases per 100 000 women and a mortality rate of 43.4 cases per 100 000 women.^7^ Screening is typically conducted using visual inspection with acetic acid. Women with a positive result are offered treatment with an ablative technique if they have lesions (that are likely to be precancerous) in the ectocervix and there is no suspicion of cancer.

WHO previously recommended cryotherapy as the preferred ablative method for the screen-and-treat approach,^8^ because it does not require electricity, is simple to use and has proven effectiveness. However, there have been recorded challenges related to ensuring an uninterrupted supply of carbon dioxide or nitrous oxide refrigerant gas, a lengthy 11-minute treatment duration and equipment reliability issues, leading to frustration among treatment providers.^9^ Another ablative technique recommended by WHO, thermal ablation, can address the challenges of cryotherapy while offering comparable efficacy.^10^^,^^11^ Such ablation can be performed by non-specialist medical doctors and nurses; it has short treatment duration (30 seconds) and does not require a gas supply. Cases that are not eligible for ablation are usually treated with large loop excision of the transformation zone (i.e. the area of cervix undergoing metaplastic transformation), hereafter referred to as loop diathermy.^12^ This approach is also the standard of care for treating all high-grade precancer lesions in high-resourced settings.^13^ Thermal ablation, cryotherapy and loop diathermy are compared in more detail in Table 1.

**Table 1:** Comparison of treatment methods used in the economic evaluation of screen-and-treat approach for cervical cancer screening, Zambia, 2023

Method, benefits	Limitations	Proportion cured after treatment of cervical intraepithelial neoplasia grades 2 and 3 (%)
**Thermal ablation** ^2^ ^,^ ^11^ ^,^ ^14^ ^–^ ^16^		
- Portable, battery-operated options - Quick (30 seconds) - Outpatient procedure - Needs minimal training - Minimal pain - No gas supply needed	- Not suitable for large lesions (> 75% of ectocervix) or lesions extending to the endocervix	82–94
**Cryotherapy** ^2^ ^,^ ^17^ ^,^ ^18^		
- Needs minimal equipment - No electricity required - Portable - Low cost - Needs minimal training - Outpatient procedure - No anaesthesia needed - Minimal pain	- Not suitable for large lesions (> 75% of ectocervix) or lesions extending to the endocervix - Needs a supply of gases - Takes 11 minutes - Post-procedure watery discharge	77–92
**Loop diathermy** ^2^ ^,^ ^16^ ^,^ ^19^		
- Provides tissue for detailed histological analysis - Higher efficacy, especially for larger/higher-grade lesions - Can also be used for endocervical or glandular lesions	- Requires electricity - Needs additional training - Higher cost - Needs local anaesthesia - Higher risk of bleeding - Risk of cervical stenosis - Higher risk of spontaneous abortion and premature rupture of membrane in subsequent pregnancies	91–98

In low-resource settings it is important to identify the simplest, most affordable and most sustainable treatment option to be used for the screen-and-treat approach. Previous studies have evaluated the costs and cost–effectiveness of loop diathermy and cryotherapy. ^1^^,^^18^^,^^20^^–^^23^ Here we compare these treatments with thermal ablation.

## Methods

The International Agency for Research in Cancer and University Teaching Hospital, Lusaka, Zambia implemented a three-arm randomized controlled trial (RCT) between 2017 and 2023, which compared thermal ablation with cryotherapy and loop diathermy, nested within a screen-and-treat programme in Lusaka, Zambia.^16^ The RCT (ClinicalTrials.gov registration: NCT02956239) was approved by the ethics committees of the International Agency for Research on Cancer and University Teaching Hospital, Zambia.

### Screen-and-treat approach

Under the screen-and-treat programme in Lusaka, trained nurses in two public-funded screening clinics (Chipata and University Teaching Hospital) performed visual inspections with acetic acid to screen women aged 25–49 years, as per Zambia’s national screening protocol. Women with a positive result who were eligible for ablative treatment were randomly enrolled into one of three treatment groups: thermal ablation, cryotherapy or loop diathermy.^16^ We aimed to compare the financial and economic costs of the three treatment options and assess their cost–effectiveness; while financial costs refer to the monetary expenditures for the treatment services, economic costs include the costs of manpower and other resources covered by the government budget and sources other than the trial (e.g. donation).

Thermal ablation was performed using a portable machine driven by rechargeable batteries (Liger Medical, Lehi, United States of America); a double freeze technique with nitrous oxide refrigerant was used for cryotherapy; and loop diathermy was performed under local anaesthesia using an electrosurgical generator (Hayden Medical, Santa Clarita, USA). More details of the trial and its outcomes are available in our previous publications.^15^^,^^16^

We tested women recruited to the study for HPV deoxyribonucleic acid (DNA) by collecting a cervical sample using a Cervex-Brush^®^ (Rovers Medical Devices, Oss, the Netherlands) in PreservCyt^®^ medium (Hologic, Marlborough, USA) just before treatment. The Xpert^®^ HPV test (Cepheid, Sunnyvale, USA) or the BD Viper™ LT system (Becton, Dickinson and Co., Franklin Lakes, USA) was used to detect high-risk types of HPV.

All participants coming for the follow-up visit one year post-treatment were evaluated by both visual inspection with acetic acid and the HPV test. Women with persistent lesions were mostly treated by loop diathermy. We defined persistent lesions as type-specific persistence of HPV at follow-up in those positive on HPV testing before treatment, or persistent positivity on visual inspection with acetic acid among those who were negative on HPV testing before treatment. Patients with cancer suspected on visual inspection and confirmed by subsequent histopathology were managed according to stage.

### Costing method

Although the RCT was nested in Zambia’s national cervical screening programme, it required additional funding – mainly to support additional staff. In this cost–effectiveness analysis we followed the International Society for Pharmacoeconomics and Outcomes Research’s Good Research Practices reporting guidance.^24^ We used a health system perspective in cost estimation that considers direct, market-valued costs required to deliver treatment and out-of-pocket payments made by patients. We did not consider non-medical costs (e.g. travel costs) and productivity costs.

First, we estimated the cost based on actual spending for the RCT (henceforth referred to as the trial scenario). To inform policy decisions for precancerous cervical lesion treatment in routine screening services beyond the RCT, we developed a routine scenario which estimated the costs by projecting expenses if screening were to be applied and integrated into routine services and replicated in facilities beyond the Chipata and University Teaching Hospital clinics. We developed a microcosting framework consisting of several cost variables, programme, personnel, equipment, consumable costs and laboratory testing costs, for both trial and routine scenarios (Table 2).

**Table 2:** Cost variables for trial and routine scenarios^a^ in the micro-costing framework for economic evaluation of thermal ablation, cryotherapy and loop diathermy for cervical cancer screening, Zambia, 2023

Cost variables	Definition	
	Trial scenario	Routine scenario
**Programme**		
Supervision	Costs of regular supervision, mainly by calls (airtime)	Costs of regular supervision, mainly by call (airtime), estimated at two times higher than expenses in the trial
Monitoring	Costs of regular monitoring, calculated from fuel used in visiting trial sites	Costs of regular monitoring, calculated from fuel used in visiting facilities sites, estimated at two times higher than expenses in the trial
Evaluation	Costs of evaluation, once per year	Costs of evaluation, once per year, estimated at two times higher than expenses in the trial
Health information system	Costs of: - regular internet connection - system development (including computers, software, and reporting system)	Cost of regular internet, estimated at two times higher than expenses in the trial No costs for system development
Laboratory information system	Costs of reporting system development, including computers	No costs for system development
Other capital investment	Head office rent and cost of utilities for the trial	No office rent costs
**Personnel**	Monthly salary of personnel involved in and dedicated to the trial. Data were extracted from the trial expenses	Assuming that the service would be integrated into clinic services, personnel would not be independently dedicated to the screen-and-treat programme. Costs were calculated based on motion–time spent for screening-related activities (in minutes), multiplied by salary per minute (based on Zambian health workers’ average salaries)
**Equipment**	Costs of purchasing cryotherapy, thermal ablation and loop diathermy equipment which will have repeated use (e.g. the machine, speculum and examination bed). Unit costs were calculated after adjustment for expected useful life and an annualization factor (discount rate of 3%)	Costs of purchasing cryotherapy, thermal ablation and loop diathermy equipment that can be used repeatedly or is reusable (e.g. the machine, speculum and examination bed). Unit costs were calculated after adjustment for expected useful life and an annualization factor (discount rate of 3%), divided by the expected number of uses per year
**Consumables**	Costs for purchase of goods and single-use items (e.g. examination gloves)	Costs for purchase of goods and single-use items (e.g. examination gloves)
**Laboratory testing**		
HPV testing	Expenses for HPV-DNA testing during screening	Expenses for HPV-DNA testing during screening
Histopathology	Expenses for processing and examination of tissue samples for histopathology (costs per sample)	Expenses for processing and examination of tissue samples for histopathology (costs per sample)

DNA: deoxyribonucleic acid; HPV: human papillomavirus.

^a^ The trial scenario was based on actual costs during the randomized controlled trial, while the routine scenario estimated costs by projecting the expenses if screening were to be expanded or scaled up to other facilities.

### Programme costs

We included supervision, monitoring, evaluation, health and laboratory information system development plus other capital investment under programme costs.^25^ We assumed that replicating and scaling up the approach for the routine scenario would involve facilities beyond Chipata and University Teaching Hospital, so costs for supervision, monitoring and evaluation would increase. We therefore arbitrarily doubled the costs for supervision, monitoring and evaluation for this routine scenario. Since the health and laboratory information systems were developed specially for the trial scenario, we did not include these costs for the routine scenario.

### Personnel costs

There were considerable differences between the two scenarios when calculating salaries. For the trial scenario, we included the monthly salary of personnel dedicated to the trial (based on expenses in the trial’s financial report) in the costing analysis. For the routine scenario we assumed that health workers would receive their regular salary from the government and would not receive a salary from the trial, and that they would not receive additional payment for performing screening and/or treatment. For this routine scenario we calculated personnel costs by assessing motion–time spent on providing services during the trial (in minutes). During our motion–time observations, we recorded the time that personnel spent on counselling, obtaining consent, examining participants, writing records, consulting with supervisors and explaining results to patients. To assess motion–time we selected women coming to the two study clinics at random and followed their motion from registration (i.e. general counselling, enrolment, collection of sociodemographic information and data entry) through to the screening examination (which includes room preparation after each examination) and treatment. Across both sites, we observed that community educators conducted 80% of the registration process and research assistants conducted the remaining registration. Nurses conducted medical history taking, screening examination, cryotherapy or thermal ablation and data entry. At both sites most loop diathermies were performed by nurses; a medical doctor only treated complicated cases. Finally, we interviewed medical staff and community educators to determine the time spent on other activities which could not be captured by motion–time observation, such as room preparation between visits, health education talks, disinfecting instruments and equipment.

To estimate personnel costs for the routine scenario, we collected the monthly average salary of different health workers, for example Zambian Kwacha (ZK) 4631 (171 United States dollars (US$)) per month for a nurse, and ZK 4320 (US$ 160) per month for a research assistant. To adjust for the change in salary before data collection and reporting, we adjusted the salary using the 2023 inflation rate. With an estimated 240 working days per year after adjustment, with 20 days of leave and holiday and eight hours per day, we estimated the salary per minute. We then multiplied the salary per minute by the time (in minutes) spent providing screening services.

### Equipment costs

We obtained information on the useful life of equipment from the manufacturer’s brochure, leaflet or the internet. We adjusted the equipment costs by spreading them over their useful life (*n*) using a 3% annual discount rate (r). We calculated the current actual cost using a process called annualization, where the annualization factor = *r*/(1−[1+*r*]*^n^*). This process helps reflect the yearly cost of equipment over its expected lifespan, rather than counting the full cost in a single year.

### Costs of consumables and testing

We collected details on costs for purchase of goods and single-use items (for example, examination gloves) from the project. We obtained costs of HPV testing and histopathology from the University Teaching Hospital laboratory.

### Data collection and analysis

We collected and managed data using the Research Electronic Data Capture web application (REDCap; Vanderbilt University, Nashville, USA).^26^ In addition to scenarios, we calculated the costs in two different terms: financial and economic. Financial costs included all expenses incurred for the trial (in the trial scenario) and all expenses at government and other facilities (in the routine scenario). However, some screen-and-treat costs were covered by other sources. For example, a Viper system was donated, an Xpert machine was purchased for another project, and information technology personnel were funded by the university. These additional resources were used to estimate the full economic costs.

As post-treatment follow-up is an integral part of screen-and-treat care continuum and 24% of participants in the trial did not undergo follow-up, we applied two approaches in calculating unit costs for both scenarios: per woman completing treatment (termed per screened-and-treated woman) and per woman completing treatment plus follow-up (termed per follow-up-completed woman).

### Cost–effectiveness analysis

We assessed the cost–effectiveness of thermal ablation relative to cryotherapy and loop diathermy using a decision tree model, run with R programming language (University of Auckland, Auckland, New Zealand), by comparing the incremental costs per successfully treated case. We based all parameters used in the analysis on data collected directly from this trial (online repository).^27^ The time horizon used in the analysis, that is, the duration over which costs and health effects are measured and compared, was one year; this corresponds to the outcome measurement one year after the treatment.

## Results

**Fig. 1:**
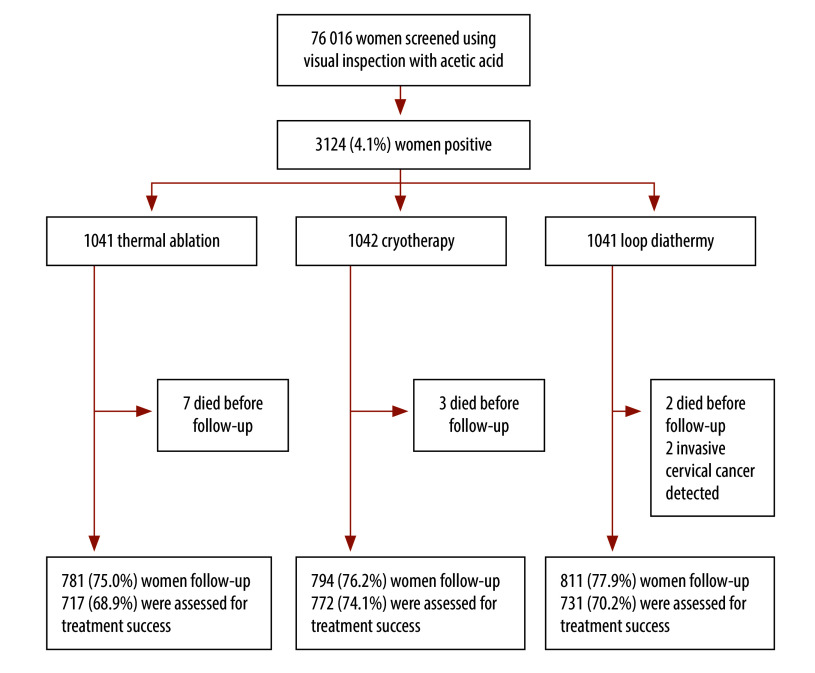
Flowchart of screen-and-treat participants in the economic evaluation of thermal ablation, cryotherapy and loop diathermy for cervical cancer screening, Zambia, 2023

A total of 76 016 women were screened using visual inspection with acetic acid at the Chipata and University Teaching Hospital clinics between August 2017 and June 2023. Of those screened, 3124 women (4.1%) with positive results following visual inspection were recruited to this study. They were assigned to one of three treatment arms (thermal ablation, cryotherapy, and loop diathermy). Out of the 3124 women who were screened-and-treated, a total of 2386 (76.4%) across the three treatment arms completed follow-up (Fig. 1).

### Motion–time

**Fig. 2:**
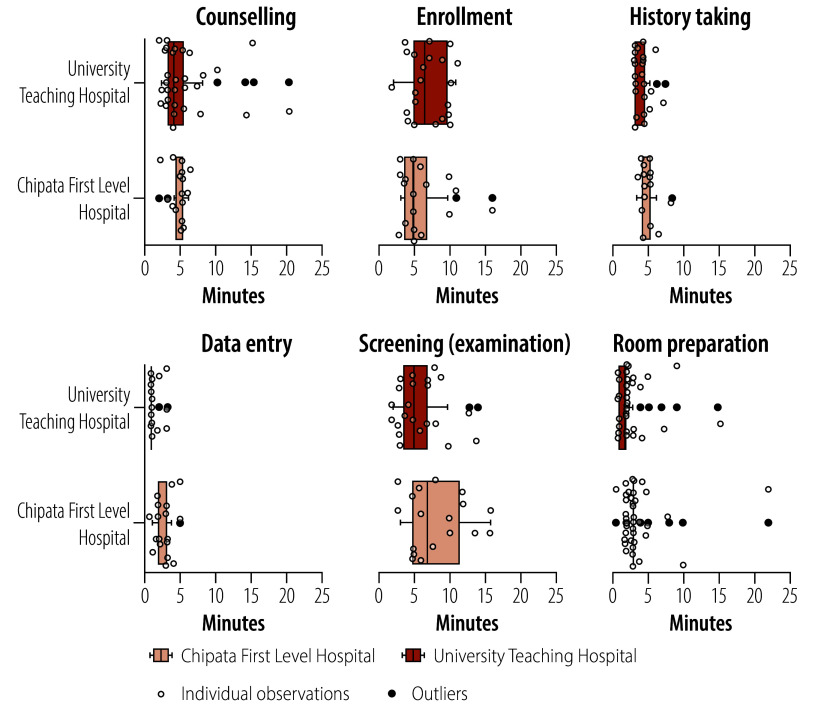
Time spent before pre-treatment counselling and treatment, by health-care facility, in the economic evaluation of thermal ablation, cryotherapy or loop diathermy for cervical cancer screening, Zambia, 2023

Note: Screening procedures included counselling, enrolment, history taking, data entry, a screening exam and room preparation for selected participants before they were assigned to the three treatment arms. Box plots show the median and interquartile range of the observed times.

**Fig. 3:**
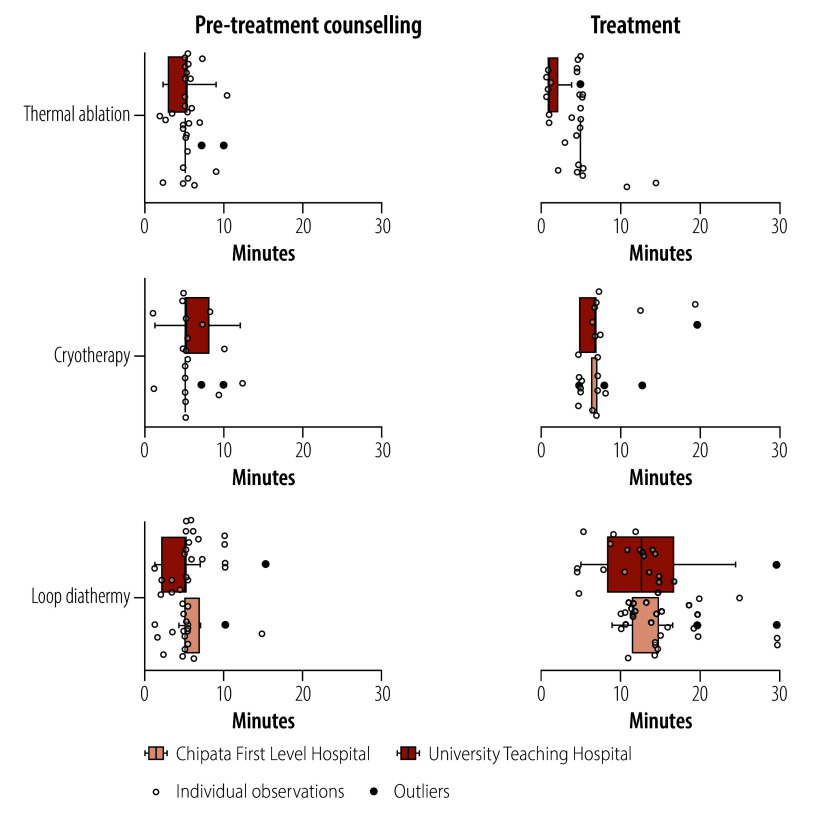
Time spent for pre-treatment counselling and treatment, by treatment type, in the economic evaluation of thermal ablation, cryotherapy and loop diathermy for cervical cancer screening, Zambia, 2023

Note: Box plots show the median and interquartile range of the observed times.

We noted motion–time differences between the two clinics. The average time for the entire screening visit was 168 minutes in University Teaching Hospital versus 124 minutes in Chipata. Fig. 2 and Fig. 3 show plots of observed times from the time and motion visits. Fig. 2 shows a breakdown of personnel time spent on various activities before pre-treatment counselling and treatment, while Fig. 3 shows time spent on pre-treatment counselling and treatment using the three techniques in the two facilities. The average time spent on loop diathermy (86 and 74 minutes in Chipata and University Teaching Hospital, respectively) was higher than time spent on cryotherapy (37 and 31 minutes, respectively) or thermal ablation (44 and 30 minutes, respectively; Fig. 3). Details of the time spent on screen-and-treat activities by different categories of staff are provided in the online repository.^27^

### Screen-and-treat costs

The financial costs of thermal ablation were the lowest compared to cryotherapy and loop diathermy in the trial scenario, for both per screened-and-treated woman (approximately US$ 39.6 versus US$ 42.3 versus US$ 50.6) and per follow-up-completed woman (approximately US$ 55.1 versus US$ 57.9 versus US$ 66.2; Table 3; online repository).^27^ The economic costs of thermal ablation were also consistently lower than cryotherapy and loop diathermy in the trial scenario. The financial costs of all treatments were almost half the economic costs in the trial, due to payment of personnel salaries by other projects and donation of a Viper system for HPV detection leading to cost-saving. In the trial scenario, personnel costs accounted for most expenses (online repository).^27^

**Table 3:** Treatment costs for each scenario^a^ and approach in the economic evaluation of thermal ablation, cryotherapy and loop diathermy for cervical cancer screening, Zambia, 2023

Costs	US$ (SD)							
	Thermal ablation			Cryotherapy			Loop diathermy	
	Financial mean	Economic mean		Financial mean	Economic mean		Financial mean	Economic mean
**Trial scenario**								
Costs per screened-and-treated woman^b^	39.6 (3.0)	75.2 (5.3)		42.3 (3.4)	78.1 (5.4)		50.6 (3.7)	94.9 (6.7)
Costs per follow-up-completed woman^c^	55.1 (3.9)	105.4 (8.5)		57.9 (4.3)	108.3 (8.5)		66.2 (5.2)	133.7 (10.9)
**Routine scenario**								
Costs per screened-and-treated woman^b^	12.7 (1.0)	13.6 (1.0)		15.6 (1.3)	16.6 (1.3)		34.9 (2.9)	35.9 (3.0)
Costs per follow-up-completed woman^c^	13.5 (1.4)	14.8 (1.2)		16.4 (1.2)	17.8 (1.3)		35.7 (3.1)	37.1 (3.1)

SD: standard deviation.

^a^ The trial scenario was based on actual costs during the randomized controlled trial, while the routine scenario estimated costs by projecting the expenses if screening were to be expanded or scaled up to other facilities.

^b^ All women screened-and-treated.

^c^ Only women that completed the follow-up visit.

Our study suggests that personnel costs would be much lower for the routine scenario (integrated into clinical services), than for the trial scenario. In the routine scenario, the cost of thermal ablation was estimated to be around US$ 13 per screened-and-treated woman and US$ 14 per follow-up-completed woman (Table 3). The financial and economic costs of the HPV test were approximately US$ 13 and US$ 26, respectively.

### Cost–effectiveness of thermal ablation

**Fig. 4:**
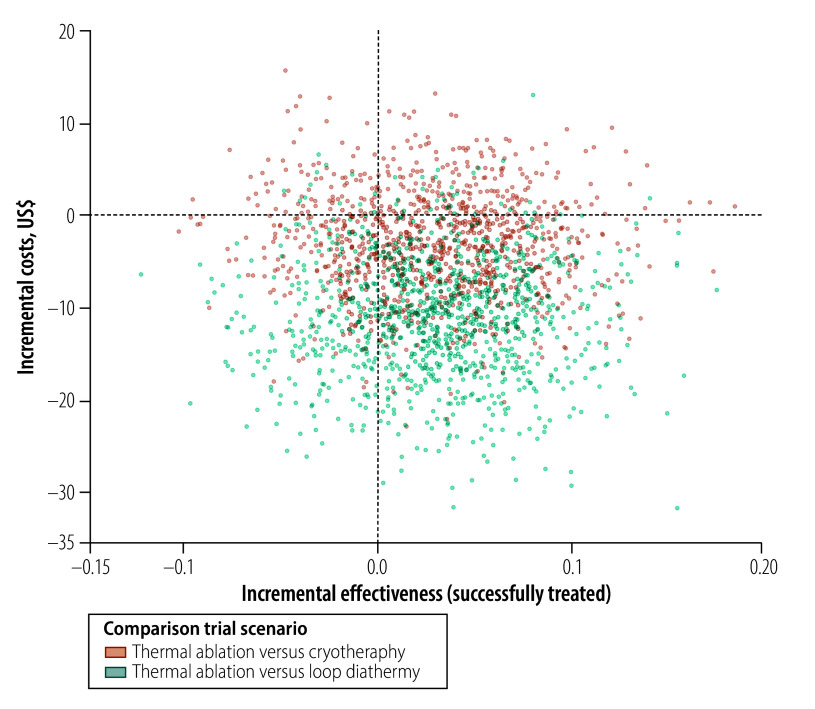
Comparison of the cost–effectiveness of treatment under a trial scenario, in the economic evaluation of thermal ablation, cryotherapy and loop diathermy for cervical cancer screening, Zambia, 2023

**Fig. 5:**
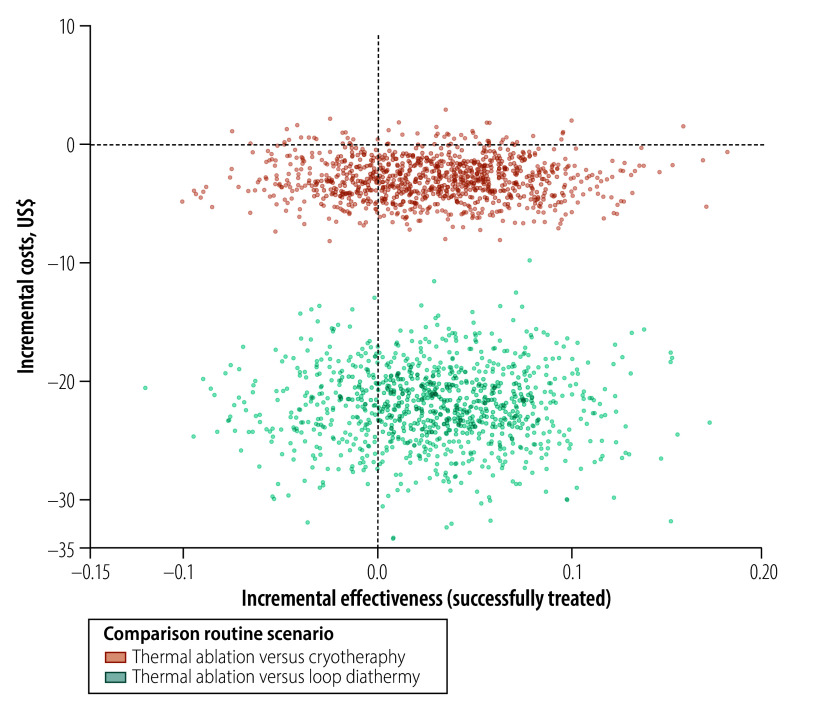
Comparison of the cost–effectiveness of treatment under a routine scenario, in the economic evaluation of thermal ablation, cryotherapy and loop diathermy for cervical cancer screening, Zambia, 2023

Following a simulation across the three groups until the end of follow-up, for the trial scenario we found that thermal ablation was cost-effective compared to cryotherapy and loop diathermy. Thermal ablation would be also cost-effective for routine use, offering a saving of US$ 29.76 per successfully treated woman over cryotherapy, and a saving of US$ 1594.99 per successfully treated woman over loop diathermy (Table 4). Fig. 4 and Fig. 5 show the incremental cost–effectiveness ratios for trial (Fig. 4) and routine (Fig. 5) scenarios, which were produced from a simulation comparing the cost and effectiveness (success) of thermal ablation compared with cryotherapy and loop diathermy. Thermal ablation had lower costs than cryotherapy and loop diathermy (Fig. 4 and Fig. 5) and it offered similar effectiveness to the other two treatments for cervical lesions (Table 4).

**Table 4:** Cost–effectiveness per 100 follow-up-completed women, in US$, in the economic evaluation of thermal ablation, cryotherapy and loop diathermy for cervical cancer screening, Zambia, 2023

Strategy	Mean cost, US$ (SD)	Effectiveness, % of successfully treated (SD)	Incremental cost–effectiveness ratio (SD)
**Trial scenario**			
Thermal ablation	54.91 (3.81)	74.0 (3.26)	Reference
Cryotherapy	57.84 (4.29)	71.1 (3.27)	−125.03 (1 850.82)
Loop diathermy	66.1 (5.12)	71.2 (3.16)	−1 557.73 (56 232.36)
**Routine scenario**			
Thermal ablation	13.44 (1.19)	74.0 (3.26)	Reference
Cryotherapy	16.38 (1.44)	71.1 (3.27)	−29.76 (1 052.92)
Loop diathermy	35.63 (3.01)	71.2 (3.16)	−1 594.99 (64 321.36)

SD: standard deviation.

## Discussion

Our study suggests that thermal ablation is a cost-effective treatment for ectocervical lesions in a screen-and-treat setting in Zambia, compared to both cryotherapy and loop diathermy. With the demonstrated high acceptability of thermal ablation among women,^28^ our study can inform the potential scale-up of the technology in low-resource settings.

Our study provides cost data for both trial and routine scenarios. Trial scenario costs were much higher than routine scenario costs due to higher personnel and research expenses. However, once established, thermal ablation would be significantly cheaper in routine service, costing nearly one quarter of the expenses required per follow-up-completed woman for the trial scenario. The reduced expense associated with thermal ablation makes it a more financially feasible option compared to cryotherapy or loop diathermy. This advantage complements previously known clinical and technical benefits.^29^ Compared to cryotherapy, thermal ablation results in fewer side-effects and offers a quicker,^29^ easier-to-perform method with simpler sterilization procedures,^30^ and no need for heavy gas cylinders, all of which enhance its acceptability.^2^^,^^14^^,^^18^^,^^30^

Although our motion–time observations suggest that pre-treatment counselling is costlier for thermal ablation than for cryotherapy, we would expect this cost to decrease as thermal ablation becomes a routine procedure and health workers become familiar with the technique. For routine service, personnel costs for thermal ablation would be much lower than for loop diathermy as the latter demands more specialized expertise and also takes longer. Thermal ablation’s simpler technique also offers advantages for knowledge transfer through trainings to large numbers of health workers – this is an important factor given the potentially high turnover rate of health workers in low- and middle-income countries.^31^

Our study has several limitations. As biopsies were only performed for loop diathermy, we could not assess overtreatment (i.e. treatment in the absence of any cervical precancerous lesions) and consequential harms following thermal ablation. We also could not assess the long-term consequences (and associated costs) of treatment with loop diathermy, such as higher risk of spontaneous abortion or premature rupture of membranes, as the evaluation period was only one year. However, as the overtreatment rate would be similar across treatment methods, and loop diathermy has a higher long-term risk of adverse reproductive outcomes compared to other treatments,^32^ thermal ablation would be both safer and more cost-effective than loop diathermy.

Although we show that HPV testing may cost up to US$ 26 per woman, leading to high upfront costs to programmes, there will be significant resource saving in the long run in switching from visual inspection with acetic acid to HPV testing, due to the prevention of a higher number of cervical cancers and reduction in screening frequency.^33^ Use of thermal ablation to treat HPV-positive women in a screen-and-treat setting will further add to resource saving.^34^

Despite the trial being nested in the Zambian cervical screening programme and making use of many routine care resources, any translation into routine service needs further careful assessment. While thermal ablation is relatively easy to perform, expertise is still needed to conduct the procedure effectively at scale. As we did not estimate costs associated with scaling up (which would involve training health workers and investing in resources) a budget impact analysis of the scaling up is also necessary. Another limitation of our study is that our cost–effectiveness analysis was limited to the trial period. We did not model long-term costs and effectiveness using outcome measures such as quality-adjusted life years, and the within-trial cost–effectiveness analysis may not have captured underlying or unobserved costs and effects.^28^ Also, we did not consider women’s perspectives separately, as societal costs are likely to be similar across the three treatment arms and minimal for healthy women undergoing screening.

Overall, our study provides evidence supporting thermal ablation as a cost-effective option for the screen-and-treat approach to cancer screening. This method could also offer a cost-effective solution for low- and middle-income countries aiming to achieve the 90% target of treatment coverage by 2030 to eliminate cervical cancer.^35^

## References

[1] Wentzensen N, Chirenje ZM, Prendiville W. Treatment approaches for women with positive cervical screening results in low-and middle-income countries. Prev Med. 2021 Mar;144:106439. 10.1016/j.ypmed.2021.106439 33678236

[2] WHO guideline for screening and treatment of cervical pre-cancer lesions for cervical cancer prevention. 2nd ed. Geneva: World Health Organization; 2021. Available from: https://www.ncbi.nlm.nih.gov/books/NBK572321/ [cited 2025 Jun 20]. 34314129

[3] Kiptoo S, Otieno G, Tonui P, Mwangi A, Orango E, Itsura P, et al. Loss to follow-up in a cervical cancer screening and treatment program in western Kenya. J Glob Oncol. 2018 Oct 1;4 Supplement 2:97s–97s. 10.1200/jgo.18.41300

[4] Basu P, Meheus F, Chami Y, Hariprasad R, Zhao F, Sankaranarayanan R. Management algorithms for cervical cancer screening and precancer treatment for resource-limited settings. Int J Gynaecol Obstet. 2017 Jul;138(S1) Suppl 1:26–32. 10.1002/ijgo.12183 28691336

[5] Khozaim K, Orang’o E, Christoffersen-Deb A, Itsura P, Oguda J, Muliro H, et al. Successes and challenges of establishing a cervical cancer screening and treatment program in western Kenya. Int J Gynaecol Obstet. 2014 Jan;124(1):12–8. 10.1016/j.ijgo.2013.06.035 24140218

[6] Lee F, Bula A, Chapola J, Mapanje C, Phiri B, Kamtuwange N, et al. Women’s experiences in a community-based screen-and-treat cervical cancer prevention program in rural Malawi: a qualitative study. BMC Cancer. 2021 Apr 22;21(1):428. 10.1186/s12885-021-08109-8 33882885 PMC8061221

[7] Sung H, Ferlay J, Siegel RL, Laversanne M, Soerjomataram I, Jemal A, et al. Global cancer statistics 2020: GLOBOCAN estimates of incidence and mortality worldwide for 36 cancers in 185 countries. CA Cancer J Clin. 2021 May;71(3):209–49. 10.3322/caac.21660 33538338

[8] WHO guidelines for screening and treatment of precancerous lesions for cervical cancer prevention. Geneva: World Health Organization; 2013. Available from: https://www.ncbi.nlm.nih.gov/books/NBK195239/ [cited 2024 Apr 18]. 24716265

[9] Maza M, Schocken CM, Bergman KL, Randall TC, Cremer ML. Cervical precancer treatment in low- and middle-income countries: a technology overview. J Glob Oncol. 2016 Aug 17;3(4):400–8. 10.1200/JGO.2016.003731 28831448 PMC5560450

[10] Dolman L, Sauvaget C, Muwonge R, Sankaranarayanan R. Meta-analysis of the efficacy of cold coagulation as a treatment method for cervical intraepithelial neoplasia: a systematic review. BJOG. 2014 Jul;121(8):929–42. 10.1111/1471-0528.12655 24597779

[11] Randall TC, Sauvaget C, Muwonge R, Trimble EL, Jeronimo J. Worthy of further consideration: an updated meta-analysis to address the feasibility, acceptability, safety and efficacy of thermal ablation in the treatment of cervical cancer precursor lesions. Prev Med. 2019 Jan;118:81–91. 10.1016/j.ypmed.2018.10.006 30342109

[12] Bigrigg A, Haffenden DK, Sheehan AL, Codling BW, Read MD. Efficacy and safety of large-loop excision of the transformation zone. Lancet. 1994 Jan 1;343(8888):32–4. 10.1016/S0140-6736(94)90881-8 7905048

[13] Debeaudrap P, Sobngwi J, Tebeu PM, Clifford GM. Residual or recurrent precancerous lesions after treatment of cervical lesions in human immunodeficiency virus-infected women: a systematic review and meta-analysis of treatment failure. Clin Infect Dis. 2019 Oct 15;69(9):1555–65. 10.1093/cid/ciy1123 30602038 PMC6792085

[14] WHO guidelines for the use of thermal ablation for cervical pre-cancer lesions. Geneva: World Health Organization; 2019. Available from: https://iris.who.int/handle/10665/329299 [cited 2024 Apr 18]. 31661202

[15] Basu P, Mwanahamuntu M, Pinder LF, Muwonge R, Lucas E, Nyambe N, et al. A portable thermal ablation device for cervical cancer prevention in a screen-and-treat setting: a randomized, noninferiority trial. Nat Med. 2024 Sep;30(9):2596–604. 10.1038/s41591-024-03080-w 38918630 PMC11405263

[16] Pinder LF, Parham GP, Basu P, Muwonge R, Lucas E, Nyambe N, et al. Thermal ablation versus cryotherapy or loop excision to treat women positive for cervical precancer on visual inspection with acetic acid test: pilot phase of a randomised controlled trial. Lancet Oncol. 2020 Jan;21(1):175–84. 10.1016/S1470-2045(19)30635-7 31734069 PMC6946855

[17] Sauvaget C, Muwonge R, Sankaranarayanan R. Meta-analysis of the effectiveness of cryotherapy in the treatment of cervical intraepithelial neoplasia. Int J Gynaecol Obstet. 2013 Mar;120(3):218–23. 10.1016/j.ijgo.2012.10.014 23265830

[18] Paul P, Winkler JL, Bartolini RM, Penny ME, Huong TT, Nga T, et al. Screen-and-treat approach to cervical cancer prevention using visual inspection with acetic acid and cryotherapy: experiences, perceptions, and beliefs from demonstration projects in Peru, Uganda, and Vietnam. Oncologist. 2013;18(12):1278–84. 10.1634/theoncologist.2013-0253 24217554 PMC3868422

[19] Jiang YM, Chen CX, Li L. Meta-analysis of cold-knife conization versus loop electrosurgical excision procedure for cervical intraepithelial neoplasia. Onco Targets Ther. 2016 Jun 29;9:3907–15. 10.2147/OTT.S108832 27418835 PMC4934869

[20] Lince-Deroche N, van Rensburg C, Roseleur J, Sanusi B, Phiri J, Michelow P, et al. Costs and cost-effectiveness of LEEP versus cryotherapy for treating cervical dysplasia among HIV-positive women in Johannesburg, South Africa. PLoS One. 2018 Oct 11;13(10):e0203921. 10.1371/journal.pone.0203921 30308014 PMC6181291

[21] Zimmermann MR, Vodicka E, Babigumira JB, Okech T, Mugo N, Sakr S, et al. Cost-effectiveness of cervical cancer screening and preventative cryotherapy at an HIV treatment clinic in Kenya. Cost Eff Resour Alloc. 2017 Jul 14;15(1):13. 10.1186/s12962-017-0075-6 28725164 PMC5513032

[22] Campos NG, Lince-Deroche N, Chibwesha CJ, Firnhaber C, Smith JS, Michelow P, et al. Cost-effectiveness of cervical cancer screening in women living with HIV in South Africa: a mathematical modeling study. J Acquir Immune Defic Syndr. 2018 Oct 1;79(2):195–205. 10.1097/QAI.0000000000001778 29916959 PMC6143200

[23] Korn AK, Muzingwani L, O’Bryan G, Ensminger A, Boylan AD, Kafidi EL, et al. Cervical cancer screening and treatment, HIV infection, and age: program implementation in seven regions of Namibia. PLoS ONE. 2022 Feb 16;17(2):e0263920. 10.1186/s13012-023-01282-3 35171941 PMC8849510

[24] Ramsey SD, Willke RJ, Glick H, Reed SD, Augustovski F, Jonsson B, et al. Cost-effectiveness analysis alongside clinical trials II-An ISPOR Good Research Practices Task Force report. Value Health. 2015 Mar;18(2):161–72. 10.1016/j.jval.2015.02.001 25773551

[25] Shin MB, Oluoch LM, Barnabas RV, Baynes C, Fridah H, Heitner J, et al. Implementation and scale-up of a single-visit, screen-and-treat approach with thermal ablation for sustainable cervical cancer prevention services: a protocol for a stepped-wedge cluster randomized trial in Kenya. Implement Sci. 2023 Jun 26;18(1):26. 10.1186/s13012-023-01282-3 37365575 PMC10294443

[26] Harris PA, Taylor R, Thielke R, Payne J, Gonzalez N, Conde JG. Research electronic data capture (REDCap)–a metadata-driven methodology and workflow process for providing translational research informatics support. J Biomed Inform. 2009 Apr;42(2):377–81. 10.1016/j.jbi.2008.08.010 18929686 PMC2700030

[27] Fuady A, Kasempa C, Lucas E, Nyambe N, Rao DW, Tenet V, et al. Supplementary of economic evaluation of thermal ablation, cryotherapy and loop diathermy in Zambia [online repository]. London: Figshare; 2025. 10.6084/m9.figshare.29314016 PMC12399993 40900929

[28] Mungo C, Osongo CO, Ambaka J, Randa MA, Omoto J, Cohen CR, et al. Safety and acceptability of thermal ablation for treatment of human papillomavirus among women living with HIV in western Kenya. JCO Glob Oncol. 2020 Jul;6(6):1024–33. 10.1200/GO.20.00035 32634066 PMC7392781

[29] Mwanahamuntu M, Kapambwe S, Pinder LF, Matambo J, Chirwa S, Chisele S, et al. The use of thermal ablation in diverse cervical cancer “screen-and-treat” service platforms in Zambia. Int J Gynaecol Obstet. 2022 Apr;157(1):85–9. 10.1002/ijgo.13808 34197624

[30] Campbell C, Kafwafwa S, Brown H, Walker G, Madetsa B, Deeny M, et al. Use of thermo-coagulation as an alternative treatment modality in a ‘screen-and-treat’ programme of cervical screening in rural Malawi. Int J Cancer. 2016 Aug 15;139(4):908–15. 10.1002/ijc.30101 27006131 PMC5084797

[31] White HL, Meglioli A, Chowdhury R, Nuccio O. Integrating cervical cancer screening and preventive treatment with family planning and HIV-related services. Int J Gynaecol Obstet. 2017 Jul;138(S1) Suppl 1:41–6. 10.1002/ijgo.12194 28691337

[32] Athanasiou A, Veroniki AA, Efthimiou O, Kalliala I, Naci H, Bowden S, et al. Comparative effectiveness and risk of preterm birth of local treatments for cervical intraepithelial neoplasia and stage IA1 cervical cancer: a systematic review and network meta-analysis. Lancet Oncol. 2022 Aug;23(8):1097–108. 10.1016/S1470-2045(22)00334-5 35835138 PMC9630146

[33] Bouvard V, Wentzensen N, Mackie A, Berkhof J, Brotherton J, Giorgi-Rossi P, et al. The IARC perspective on cervical cancer screening. N Engl J Med. 2021 Nov 11;385(20):1908–18. 10.1056/NEJMsr2030640 34758259 PMC12125667

[34] Toliman PJ, Kaldor JM, Tabrizi SN, Vallely AJ. Innovative approaches to cervical cancer screening in low- and middle-income countries. Climacteric. 2018 Jun;21(3):235–8. 10.1080/13697137.2018.1439917 29488817

[35] Global strategy to accelerate the elimination of cervical cancer as a public health problem. Geneva: World Health Organization; 2020. Available from: https://www.who.int/publications/i/item/9789240014107 cited 2025 Jun 24].

